# Neuron participation in a synchrony-encoding assembly

**DOI:** 10.1186/1471-2202-7-72

**Published:** 2006-10-27

**Authors:** Florence Duret, Svetlana Shumikhina, Stéphane Molotchnikoff

**Affiliations:** 1 GREN, Université Catholique de Louvain, Belgique; 2 Département de Sciences Biologiques, Université de Montréal, CP 6128 Succ. centre-ville, Montréal, PQ, H3C 3J7, Canada

## Abstract

**Background:**

Synchronization of action potentials between neurons is considered to be an encoding process that allows the grouping of various and multiple features of an image leading to a coherent perception. How this coding neuronal assembly is configured is debated. We have previously shown that the magnitude of synchronization between excited neurons is stimulus-dependent. In the present investigation we compare the levels of synchronization between synchronizing individual neurons and the synchronizing pool of cells to which they belong.

**Results:**

Even though neurons belonged to their respective pools, some cells synchronized for all presented stimuli while others were rather selective and only a few stimulating conditions produced a significant synchronization. In addition the experiments show that one synchronizing pair rarely replicates the level of synchrony between corresponding groups of units. But when synchronizing clusters of neurons increase in number, the correlation (measured as a coefficient of determination) between unit synchronization and the synchronization between the entire pools of cells to which individual neurons belong improves.

**Conclusion:**

These results prompt the hypothesis that random or spontaneous synchronization becomes progressively less important, whereas coincident spikes related to encoding properties of targets gain significance because a particular configuration of an image biases the excitatory inputs in favor of connections driven by the applied features of the stimulus.

## Background

Over the past several decades, more than twenty different cortical areas have been recognized as processing loci of specific properties of visual scenes, such as form, color, motion, etc. In fact, new brain imagery techniques assign particular functions to specific cortical sites. It remains to be demonstrated how these distributed activities are combined to provide coherent visual targets. Certainly, electrophysiological investigations carried out on monkeys pointed out few locations in the nervous system where the parcellated information of visual scenes and objects could be resynthesized to form a unified and a coherent percept (see review [1]). In these previous investigations, it was shown that the cellular firing rate may suffice to encode image properties. But a different view also emerged: it has been suggested that synchronized firing between excited cells allows linkage ofimage properties. For instance, it has been proposed that synchronization of action potentials, within a time-window of 1 to 5 ms, between two or more neurons belonging to distant pools of cells may be an encoding process, permitting the binding of various features of a single visual object [2-8], but see the review by Logothetis & Sheinberg, 1996, [9]. Such a binding could arise whether local properties are similar or dissimilar. For example, when a group of cells are excited with two collinear light bars, they may fire in synchrony. But, it has also been shown that synchronization of action potentials happens when orthogonal angles are formed in an image. Such linking of image properties by synchronizing action potentials has been defined as a coding assembly [10-15] or synchrony-encoding assembly. Hence, binding through synchrony of action potentials, in general terms, is a process permitting the functional linkage of distributed neuronal activity [16,17]. Interestingly, it has been previously reported [11,15,18] that synchronization modulations are unrelated to firing rate increases resulting from co-excitation of a common input determined by the same stimulus.

Numerous previous studies have been carried out with multiunit recordings where several cells are recorded simultaneously. Although suggestive of the activity of a neuronal network, this method fails to identify which cells participate in the synchronization process and consequently how an encoding assembly is formed, [19,20]. Hence, when slight modifications are introduced in image configurations, the participation of cells in the synchrony-encoding assembly changes. However, do all units contribute equally to the formation of the synchrony-encoding assembly or do some units join or leave the grouping as local features of the image change during natural viewing? Indeed, recently we demonstrated that a high degree of synchronization between multiunit discharges shown by cross-correlograms may mask the contribution of individual cells [10,11].

The present investigation aims to further understand the processes leading to the formation of encoding assemblies by synchronization. For this objective we adopted our previous paradigm for target presentations [11] which showed that synchrony magnitude may be modulated when two sine-wave grating patches are laterally displaced in the visual field. In these configurations one central patch covers both receptive fields while a second patch, called contextual, is displaced laterally. Both patches share identical properties such as contrast, spatial and temporal frequencies and velocity. Thus the only aspect which distinguishes one image from another is the distance between both stimuli, i.e., the spatial relationships. We investigated the synchronization between pairs of neurons sorted out from multiunit recordings. Results revealed that the level of synchronization of one pair of cells fails to reflect the synchrony-encoding assembly of the pool of neurons. The consequence of this last result could mean that when considering one pair of cells, the spontaneous synchronization may be high enough to mask stimulus-related-synchronization, whereas when other pairs of cells are added to the neuronal assembly, random synchronization is progressively outweighed, and the pattern of stimulus induced synchronization is revealed.

## Results

**Figure 1:**
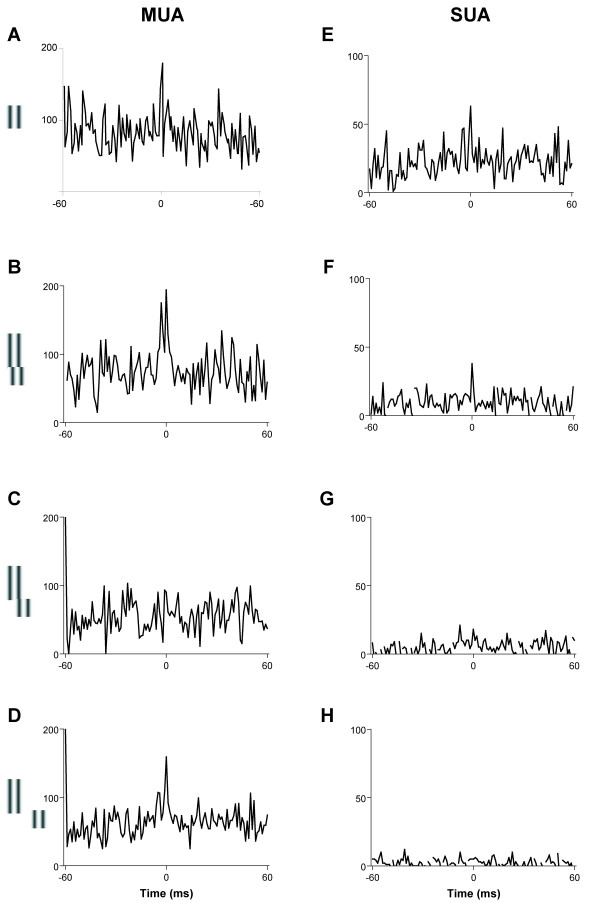
Comparison of cross-correlograms (XCRG) between multi-unit activity – left- (MUA) and single unit activity – right- (SUA). The sine-wave patches configuration is shown on the left side of XCRGs. In A only the CRF is stimulated in isolation with one patch. In B-D the added lower patch is shifted laterally by 0.5, 4, 8 deg. respectively. Notice that in SUA the number of spikes is lower.

**Figure 2:**
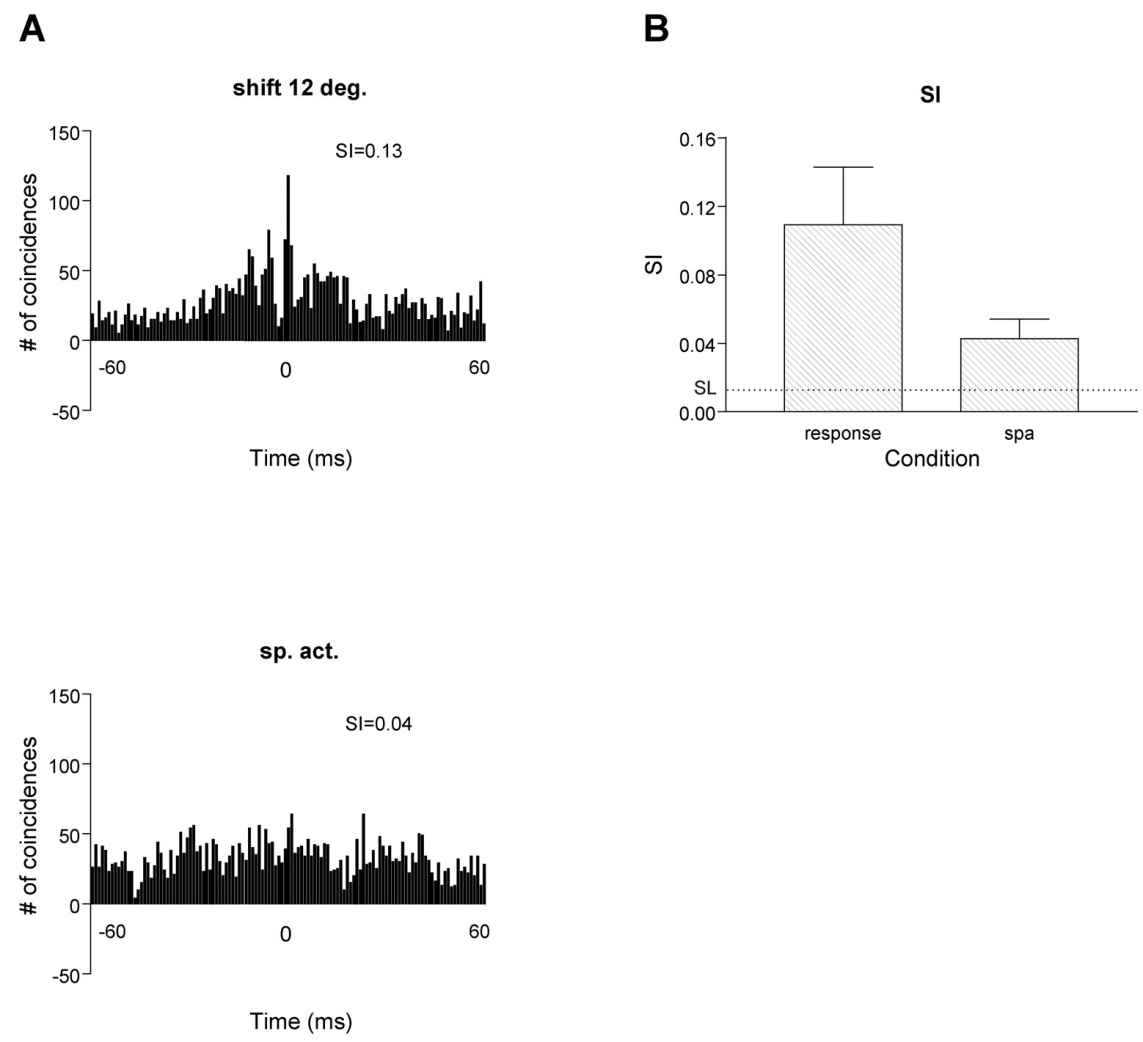
Comparison of cross-correlograms between evoked responses and spontaneous activity. A upper: the lower patch of the stimulus (see figure 1) is shifted by 12 deg. The cross-correlation produced a synchronization index: SI = 0.13. A lower: spontaneous activity (sp.act.) no significant synchronization SI = 0.04. B: Averaged synchronization index. Evoked responses (response) and spontaneous activity (spa). Visual stimulation noticeably enhances cortical synchrony. SL: significance level.

The study was carried out in areas 17 and 18 of cats. The analyses were performed on 12 sites yielding 54 multiunit recordings. The synchronization index (SI) or synchronization strength was statistically significant in 53 cases for at least one stimulus configuration. We will refer to these pools of neurons as "multiunit data." Cross-correlating single unit responses shows a lower incidence of synchronization. Thirty-one different neurons were extracted from the twelve recording sites. They formed 40 pairs of cells referred to as "single-unit data." Thirty pairs yielded statistically significant synchronization for at least one applied target. These cells are called 'participating cells' to synchrony-encoding assembly. Figure 1 shows one example. Cross-correlating multiunit activity (MUA) produces a central peak suggesting synchrony between both pools of neurons. The latter occurred for the following image structures: compound receptive field stimulation in isolation (A), and shifts of 0.5°, 8° (B, D), while a 4° displacement (C) of the lower patch failed to produce a synchrony (Fig. 1). Two units (SUA: single unit activity), one from each site, were sorted out from these two pools of cells and were recorded on all stimulus conditions. Cross-correlating their activities generated synchrony only for the CRF stimulation in isolation (E) and a 0.5° shift (F) while shifts of 4° (G) and 8° (H) conditions revealed an absence of time relationships, in spite of the fact that there was a central peak with the multiunit recordings (Fig. 1). The synchronization also was computed for spontaneous activity. However cross-correlograms may be carried out meaningfully only if available spikes exceed 600 per channel [21]. Since cortical cells are relatively inactive in absence of stimulation it becomes impractical to perform valuable cross-correlations within a reasonable period of time. Consequently, we cross-correlated neuronal activity when the number of spikes allowed the computation to be executed. For each pair, cross-correlations were made separately for spontaneous and stimulation periods. Weak synchrony is sometimes observed during spontaneous activity, though visual stimulation noticeably enhances (P < 0.001) cortical synchrony (Fig. 2A, B). Figure 2 illustrates an example. The number of coincident spikes in the central bin indicates a very low SI: 0.04 (Fig. 2A lower). On the other hand the stimulation produced a cross-correlation with a significant peak: SI = 0.13 (Fig. 2A upper). Figure 2B shows the average SI in spontaneous activity (spa) and when stimuli were applied (response).

### Participation in assembly

**Figure 3:**
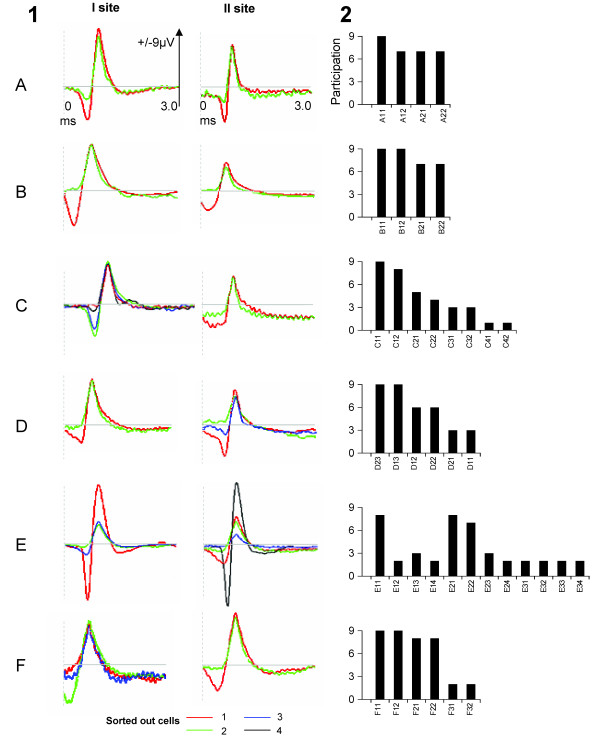
1: Participation in synchrony-encoding assembly. Examples of individual neurons sorted out from paired sites labeled A to F. In all cases Z-score > 2.5 (significant level), details in text. Total time course 3.0 ms. 2. Participation and synchronization distribution of each pair of cells. X-axis: A11 indicates responses of cell 1 from site I are cross-correlated with neuron 1 from site II; in this instance synchronization is obtained for all cases. A12 indicates that firing of cell1 from site I is cross-correlated with neuron 2 from site II; in this instance synchronization is obtained in 8 cases. Y-axis: number of configurations (including spontaneous activity, no stimulus present).

As indicated above, thirty pairs yielded statistically significant synchronization for at least one configuration and ten failed to reach significance threshold. Out of these thirty pairs of cells exhibiting synchronized firing, four presented a statistically significant SI in 10 to 50% of configurations evoking responses and twenty-six (87%, N = 30) exhibited a statistically significant synchronization in more than 50% of applied configurations (see Methods). Similar proportions were obtained with multiunit data. Six paired sites are illustrated with averaged spike waveforms sorted out from each pool of neurons as recorded simultaneously with two electrodes (5 to 10 action potentials are averaged; inter-electrode distances 400 to 800 μm) (Fig. 3 column 1). In these examples each action potential could be followed for applied configurations, and cross-correlograms were carried out between respective cells. For instance, from the examples shown in figure 3 row C, the cell labeled 1 in site I and cell 1 in site II exhibited significant synchronization to all nine applied conditions (histogram, row C, C11 on X-axis) whereas the cells labeled 4 and 1 from the same sites and recorded simultaneously synchronized only to one configuration (histogram, row C, C41 on X-axis). Hence, for some image configurations, the latter two units failed to synchronize and thus withdrew from the coding assembly. The participation histograms (column 2 Fig. 3) summarize our data. The number of configurations in response to which a particular cross-correlation yielded significant value of the central peak is indicated on the Y-axis. Quite notably, even though neurons belonged to same respective pools, these distributions reveal that some cells synchronized for all configurations while others were rather selective and only a few stimulating conditions showed a significant synchronization suggesting that synchrony-encoding assembly may be rather selective.

### Assembly formation

**Figure 4:**
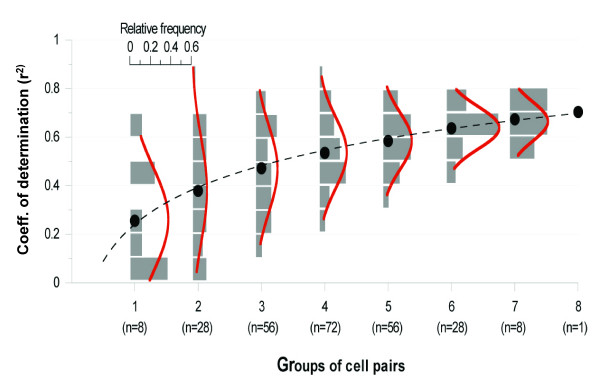
Coefficient of determination (r^2^) between the modulation of synchronization of grouped cell pairs and the synchronization changes of multi-unit data. In X-axis: groups of cell pairs, for example, group 1 means one pair. The synchronization magnitude and modulation of each pair of cells (eight values) were calculated and compared (r^2^) with changes of synchronization of multi-unit data. In both cases identical image configurations were presented. Then groups were increased in size two by two, and the same computations were carried out, that is, for twenty-eight combinations. This procedure was repeated for grouped cell pairs of 3, 4, 5, 6, 7, and 8, that is, 56, 72, 56, 28, 8, 1 combinations, respectively. The relative distribution is shown above the group size. The black dot shows the average value. Y-axis: values of r^2^. The red curve is the resulting normal distribution. More details in text.

As suggested by figure 3 the contribution of participating neurons differs from one set of stimuli to another. The next questions raised by the above results is to what extent does synchronized activity between two neurons reveal the behavior of the cluster of cells to which these units belong? To answer this question we further analyzed a group of eight pairs of cells that we were fortunate to record at both sites for all stimulus conditions. For this purpose we measured the relative strength of synchronization between groups of paired cells in response to each target structure and correlated this value with synchronization modulations measured in multiunit recordings for the identical stimulus configurations. The selected neurons of each pair belonged to their respective multiunit populations. In the first step the synchronization changes of each pair were correlated with the average synchronization modifications derived from multiunit data (that is, eight values, data point in relative frequency plot, above 1, Fig. 4). Then cell-pairs were grouped two by two (yielding 28 combinations or permutations, data point in relative frequency plot, above 2, Fig. 4). Variations of synchrony of each combination were averaged and correlated with averaged multiunit values. These computations were repeated by assembling three pairs (56 correlation points), four pairs (72 correlation points), five pairs (56 correlation points), six (28 correlation points), seven (8 correlation points), and finally all eight pairs put together. Thus, for each combination the coefficient of determination [22] with the multi-unit synchronization pattern was computed. Our results showed that when pairs of cells are assembled by two or three, they reflect the multiunit behavior poorly, since coefficients of determination values (r^2^) varied from 0.04 to 0.9 (mean = 0.38, SD = 0.22), and from 0.16 to 0.8 (mean = 0.47, SD = 0.18), respectively. In contrast, when cell-pairs were grouped by four, five, six, or seven, the synchronization modulation approached, on average, the multiunit recordings (means were 0.54, 0.58, 0.63, 0.66, and SD were 0.15, 0.12, 0.09, 0.09, respectively). Moreover, when groupings were made of seven cell-pairs, the mean of synchronization modulations of each permutation was similar to the multiunit value (smallest r^2 ^was 0.52 for only one combination and r^2 ^= 0.7 for a group of 8, Fig. 4). Such high values suggest that, as one would expect, several pairs of synchronized neurons must be measured to obtain a more accurate understanding of the process leading to a synchrony-encoding assembly. Therefore and most importantly the modulation of the synchronization strength of one pair of neurons is inadequate to deduce the synchronizing strength of the pool of cells to one particular stimulating target.

Finally histograms of figure 4 are positioned according to their group size. These distributions further indicate that the width of the distribution progressively narrowed as the number of grouped pairs of cells increased. In parallel, the SD progressively became lower, σ = 0.25, 0.22, 0.16, 0.15, 0.12, 0.09, 0.09. Finally, the normalized distribution curves (in red) are shifted to the right, indicating that the coefficients of determination values were better. However we should be cautious and keep in mind that the above measures are correlations between synchronous firing and displacement of image elements. We still need to address how the image as a whole is encoded.

## Discussion

As intuitively expected, taken together those findings suggest that with larger numbers of grouped pairs of cells, one comes close to a synchronized firing similar to the multi-unit data. Therefore, any spontaneous or accidental synchronization, which is infrequent because it happens in a very narrow time-window of a few milliseconds, carries little weight in comparison to the barrage of synchronization induced by many input fibers discharging during a time window that is established by the presence of the stimulus which generates a common input. Hence, the above data would suggest that spurious synchronization, produced randomly and observed with a small number of grouped pairs of cells, may be 'immersed' in synchronization induced by a visual stimulus and therefore, only synchronization produced by visual stimuli is salient. Indeed, since targets may share a number of characteristics (in our case distance separating the two patches with the following identical properties: orientation, drift direction, velocity and spatial and temporal frequencies), several pools of cells are activated creating a putative stream of action potentials closely related in time. Hence any accidental synchrony will be absorbed in the surge of simultaneous spikes induced by target properties. This line of reasoning may be appreciated by comparing the relative frequency between groups (Fig. 4). For instance, when a group consists of one pair, then in only one case did we measure a high coefficient of determination suggesting that cross-correlation modulations of this particular pair represent the population's cross-correlation changes. By comparison, if a single group was made up of 8 pairs and the coefficient of determination equaled 0.7, this suggests the behavior of the group reasonably reproduced the multiunit activity.

Finally, it is important to note, as we have previously demonstrated with very similar experiments [11,18], that the above modulations of the strength of synchronization are unrelated to the firing rate, because the compound receptive field is always stimulated with the same target and when the firing rates change the fluctuations are dissociated from modulations of synchronization levels. Likewise the synchronization index derived from spontaneous activity is significantly lower than the values obtained when stimuli are applied [11,15,16]. Obviously, it is impossible to record from all cells within a short distance of the electrode tip and consequently the numbers presented in this investigation underestimate the total number of units making up a coding assembly for one particular image.

In contrast, others have argued that synchronization could not be a phenomenon implicated in the sensory binding task since too many neurons are present in a small cortical area with an enormous number of synaptic connections and a high level of synchronization occurring without relationship to any particular target [19,20]. This spontaneous or accidental synchronization is always present. Indeed, it has been calculated that a typical cortical neuron receives several thousand synaptic inputs [23], but see below. Because of cortical modular organization, nearby neurons generally have similar functional properties and hence fire roughly together [24]. Visual cortical neurons excited by an appropriate stimulus usually exhibit firing rates in the range of 10–100 spikes per second. Consequently, neurons must be able to ''distinguish" random synchronizations from synchronizations that "make encoding sense" [12]. There must be physiological mechanisms that allow neurons to distinguish inputs originating from the same or different arriving cells within the matching time-windows. Moreover, temporal and spatial summations from the same afferent must be ignored and only temporally correlated firing from different neurons must be considered. In other words, as stated by several investigators [19,25], how is a neuron able to engage in selective synchronous interactions with a subset of its inputs when a fraction of all of the cell's inputs is active and synchronous? On the other hand, cortical cells may act as coincident detectors. Indeed, it has been demonstrated [26,27] that cortical neurons' responses may depend very much on the interval between two arriving spikes and any intervals less than 1 ms are a very potent reinforcement for the efficacy of synaptic transmission. Data [28-30] (see Sejnowski & Paulsen, 2006, [31]) further show that membrane properties of cortical cells are compatible with such behavior and that neurons are sensitive to time correlations. Yet the detection of two temporally correlated active synapses must take place amongst other active afferents. This constraint is partly alleviated by the recent observation that thalamocortical synapses account for fifteen percent of synapses onto a cortical neuron. In addition these synapses are weak. Hence it is suggested that cortical cells are activated by synchronous activity [32,33]. Indeed, simultaneous activation may boost post-synaptic responses. One may suggest that the effect from accidental synchrony would be small in relation to the enhancement expected from a synchronous barrage produced by stimuli that activate common inputs. Finally the above arguments do not rule out a contribution of a firing rate code as a process of signaling coherent images.

## Conclusion

At the neuronal population level encoding by synchronization may be reminiscent of a game of stacked dice or loaded dice. Even if accidental or intrinsic synchrony may be relatively common, the synchronization induced by the linkage of particular visual features of a stimulus may be functionally significant if there are a large number of synchronizing cells included in the neuronal assembly. In other words, if a phenomenon includes both random and non-random events, an exogenous driving force, which in this case is the application of the target, stimulates a larger number of units to synchronize their action potentials and random synchrony is masked as noise. By analogy, this phenomenon of synchronization behavior may be compared to stacked dice, with each face being differently weighted (corresponding to a visual configuration). With a few tosses of these dice (a few accidental synchronous spikes), a naïve player will be unable to realize that there is something wrong with the dice, since numbers obtained randomly differ little from the ones due to stacked numbers. In our synchronization models, when only few pairs of neurons participate or unexpected coincident spikes occur, the resulting synchrony is indistinguishable from synchrony attributed to spontaneous activity. However, with an increased number of tosses (i.e., more synchronizing clusters of neurons, and the neuronal assembly is enlarged), the numbers on the dice will favor certain patterns (or, by analogy, random or spontaneous firing will progressively count less, whereas coincident spikes related to encoding properties of targets will gain significance). In physiological terms, correlated activity surpasses spontaneous levels of synchrony when clusters of neurons in an assembly are activated together by visual stimuli. That is, in spite of spontaneous synchrony this particular configuration biases the afferent inputs in favor of parallel connections driven by the applied images.

## Methods

### Animal preparation

Cats (5) of 2.5 to 3.2 kg, premedicated with atravet and atropine, were anaesthetized with ketamine prior to catheterization of the forelimb vein and tracheotomy. Xylocaine was injected at surgical sites. Cats were placed in the stereotaxic apparatus, paralyzed with Flaxedil and artificially ventilated with a mixture of gases (N_2_O/O_2_: 70/30; isoflurane 0.5%) for the duration of the experiment. Flaxedil was delivered to the animal for the duration of the experiment with a mixture of 5% dextrose in lactated Ringer's solution. A heating pad was used to maintain body temperature. ECG, expired CO_2 _and EEG were monitored throughout the experiment. The end-tidal CO_2 _partial pressure was kept constant between 28 and 30 mmHg by adjusting the rate and depth of respiration. An antibacterial agent, Tribrissen, and Penlong antibiotic were administered to the animals. Pupils were dilated with atropine and the nictitating membranes were contracted with phenylephrine hydrochloride. Plano contact lenses with no artificial pupils were placed on the eyes to prevent the cornea from drying. The positions of the area centralis were inferred from the position of the blind spots, which were ophthalmoscopically back-projected onto a translucent screen. During the experiment, the unused eye was occluded with an opaque board [18,34]. At the end of the experiments, cats were euthanized with Somnotol. The experimental protocol was approved by the committee for animal care of the University of Montreal and also conforms to guidelines of the National Institutes of Health.

### Visual stimulation

After neuronal activity was obtained, the receptive field of at least two groups of cells was located using a hand-held projector with a narrow slit of light projected onto a hand-held screen placed 57 cm from the cats' eyes. Receptive field properties such as dimension, orientation and directional selectivity, and velocity preference were noted. Supplementary sine-wave patches were positioned as follows. Two drifting sine-wave gratings with properties identical to the central patch were added; they were located above and below the compound receptive field, CRF (80% contrast in all cases). These peripheral patches were outside the compound receptive field since they failed to change the firing rate when applied in isolation. In total nine conditions or configurations were used: the CRF stimulated in isolation, all patches aligned, one patch (above or below the central one) was shifted laterally in steps by 0.5, 1, 2, 4, 8, 12 deg., (from center to center) and finally the spontaneous activity. These nine configurations were randomly applied. The distance between the central patch and the one in the periphery was the unique property differentiating the targets' structures since other properties were identical (contrast, spatial and temporal frequencies, velocity). It must be emphasized that, except for spontaneous activity, the central patch was always applied, hence receptive fields were stimulated under every condition which permitted maintaining a reasonable rate of firing to carry out cross-correlograms and verify that sorted out cells were active.

### Recordings

Multiunit activity in the visual cortex was recorded by two sets of matrix tungsten microelectrodes (FHC, Inc. Frederick Haer & Co., 10 μm tip exposure, 10 MΩ each). Each set, consisting of four microelectrodes (400 μm fixed separation between the nearest microelectrodes) enclosed in stainless steel tubing, was independently connected to two micromanipulators. One set was positioned in area 17 and the second in area 18, in superficial layers. After the microelectrodes were inserted, the cortex was covered by warm agar (3–4% in saline) and wax. The neuronal action potentials were amplified and sent to a computer for voltage discrimination and recording with 0.05 ms resolution for on-line and off-line analyses (DataWave Technologies). Multiunit recordings from one electrode included usually up to 4–5 well isolated single units which were thresholded, that is, isolated from the noise. Partially overlapping receptive fields were stimulated with a patch of drifting sine-wave gratings whose properties were adjusted to evoke sufficient activities in two pools of neurons. Hence, it was necessary to find a compromise between stimulus parameters to evoke the adequate number of spikes in both trains to carry out cross-correlograms. In most cases 20 to 30 spikes per sec. is a satisfactory number of action potentials to generate valid cross-correlograms (see below).

Individual units were sorted out from within multiunit activity by a spike separation method using commercial software (Autocut, DataWave Technologies). Spike sorting is based on the assumption that action potentials from different cells have different amplitude and temporal characteristics and that these characteristics are stable during a single trial recording and across trials. Because spike separation is performed off-line attention was first focused on data acquisition. Tests were made during control recordings to insure that a time window of on-line unit extraction was sufficient to reproduce fully spike waveforms off-line. During the recordings, the action potentials were detected by their voltage threshold crossing and the unit extraction was centered on the peak of action potentials. Usually, three milliseconds of digitized voltages with a peak pre-time of 0.5–0.7 ms were sufficient to reproduce the shape of action potentials. The spike sorting procedure was performed automatically by the software using eight parameters such as amplitude (height) and width of peaks and valleys of the action potential, spike area and ratio of peaks. These principal component values (eight parameters) form clusters and the Z-score estimates the statistical significance of spike separation (Z-score had to be superior to 2.5). Elliptical cluster boundaries were used. Discriminated spikes were individually visualized and monitored along with standard deviations that ensured that the waveform of selected spikes remained within determined boundaries. The results of cluster analysis as well as isolated spikes were visually inspected by screening the clustering and superposition and average of their waveforms in the chosen time window. As an additional control, a raster plot of activity with color coded isolated spikes and histograms of auto- and cross-correlation analyses between isolated spikes was checked for possible errors of spike separation.

Then, when individual cells were extracted, cross-correlograms (XCRGs Datawave) software could be carried out, and the synchronization magnitude between the different pairs of neurons could be computed, as well as their statistical significance, as described below.

### Data analysis

Peristimulus time histograms (PSTHs) and cross-correlograms (XCRGs, bin width 1 ms) were computed. Each stimulus was repeated 20–30 times for 4096 msec. For a given recording, the number of stimulus presentations was constant. There was no lock-out time between successive spike acquisitions. In all cases the receptive fields of the recorded neurons were in the center patch. The quantitative evaluation of multi-unit responses was achieved electronically with images generated on a monitor screen (Mitsubishi Electronics) with a refresh rate of 100 Hz and centered on the compound receptive field and synchronized with the acquisition processes. During a recording session, signals from at least two electrodes were amplified and digitized (Datawave Technologies, CO, USA).

The epoch used to compute the XCRGs was chosen from PSTHs and included the portion of recordings where both responses overlapped. PSTHs (bin width 10 ms in all cases) were computed for time of analysis corresponding to the time of visual presentation. After single cells were sorted out off-line from multi-unit spike trains accumulated during data acquisition, cross-correlograms (XCRGs) were constructed. To examine synchronization of neural origin that is the induced synchrony which is unrelated to the stimulus onset, one needs to remove stimulus-induced time correlation. For this purpose, shift predictors were computed by correlating spike recordings shuffled by one or two stimulus presentations [35], and these were subtracted from the raw XCRGs to obtain the difference XCRGs ([raw minus shuffled]). These computations remove the stimulus-locked component. Hence all subsequent analyses were performed on the difference XCRGs. Synchronization strength was computed as a correlation coefficient [36-38]. This correlation coefficient, or synchronization index (SI), reflects the strength of the synchronization in a neural XCRG as a function of the number of events in the central bin normalized in relation to the firing rate of each neuron. As a consequence, the synchronization strength may be considered to be independent of the firing levels, i.e., the mean spiking rates. The synchronization index is defined as

SI = CE/{[N_1_-(N_1_^2^/T)]·[N_2_-(N_2_^2^/T)]}^1/2 ^    (1)

where CE is the number of coincident events in the central bin, and N_1 _and N_2 _are the total number of discharges recorded simultaneously from two neurons over time T (4,096 ms × number of trials) [38]. This equation determines the proportion of spikes that occurs simultaneously in a pair of cells. Because this ratio takes into account the firing rate of activity of both paired sites, it allows a direct comparison between different conditions. The magnitude of synchrony was computed only in the central bin because it allows a precision within a 1-ms range (zero-lag synchronization). In addition, such a short time-window reduces the probability of recording accidental synchrony. The significance of the peak in the XCRG is estimated by computing a Z score. If the latter is >4 the peak is considered significant. The significance level is calculated as SL = 4(t/T)^1/2 ^where t is the bin size in the XCRG (1 ms) and T is the time of recording.

## Authors' contributions

FD carried out all experiments and performed statistical analysis. SS carried out analysis and participated in experiments. SM participated in the design of the study, the data analysis and wrote the manuscript. All authors read and approved the final manuscript.
